# Social Determinants of Health and Health Equity in the Treatment and Rehabilitation of Sport-Related Concussion: A Content Analysis of Intervention Research and Call-To-Action

**DOI:** 10.1089/neu.2023.0550

**Published:** 2024-10-21

**Authors:** Nathan E. Cook, Alicia Kissinger-Knox, Ila A. Iverson, Katie Stephenson, Marc A. Norman, Amy A. Hunter, Altaf Saadi, Grant L. Iverson

**Affiliations:** ^1^ Department of Physical Medicine and Rehabilitation, Harvard Medical School, Boston, MA, USA.; ^2^ Mass General for Children Sports Concussion Program, Waltham, MA, USA.; ^3^ Department of Physical Medicine and Rehabilitation, Spaulding Rehabilitation Hospital, Charlestown, MA, USA.; ^4^ Concussion Research Program, Spaulding Hospital Cambridge, Cambridge, MA, USA.; ^5^ Department of Global Public Health, Karolinska Institutet, Stockholm, Sweden.; ^6^ College of Osteopathic Medicine, University of New England, Biddeford, ME, USA.; ^7^ Department of Psychiatry, UC San Diego School of Medicine, San Diego, CA, USA.; ^8^ Department of Public Health Sciences, University of Connecticut School of Medicine, Farmington, CT, USA.; ^9^ Department of Pediatrics, University of Connecticut School of Medicine, Farmington, CT, USA.; ^10^ Injury Prevention Center, Connecticut Children’s Medical Center and Hartford Hospital, Hartford, CT, USA.; ^11^ Department of Neurology, Harvard Medical School, Boston, MA, USA.; ^12^ Department of Neurology, Massachusetts General Hospital, Boston, MA, USA.; ^13^ Schoen Adams Research Institute at Spaulding Rehabilitation, Charlestown, MA, USA.

**Keywords:** ethnicity, mild traumatic brain injury, persistent postconcussion symptoms, race, socioeconomic status

## Abstract

This review was designed to (1) determine the extent to which the clinical science on sport-related concussion treatment and rehabilitation has considered social determinants of health (SDoH) or health equity and (2) offer recommendations to enhance the incorporation of SDoH and health equity in concussion treatment research and clinical care. The Concussion in Sport Group consensus statement (2023) was informed by two systematic reviews examining prescribed rest or exercise following concussion and targeted interventions to facilitate concussion recovery. We examined 31 studies, including 2,698 participants, from those two reviews. Race (*k* = 6; 19.4%) and ethnicity (*k* = 4; 12.9%) of the study samples were usually not reported. Four studies examined ethnicity (i.e., Hispanic), exclusively as a demographic category. Five studies (16.1%) examined race as a demographic category. Three studies (9.7%) examined socioeconomic status (SES; measured as household income) as a demographic category/sample descriptor and one study (3.2%) examined SES in-depth, by testing whether the treatment and control groups differed by SES. Five studies examined an SDoH domain in a descriptive manner and four studies in an inferential/intentional manner. No study mentioned SDoH, health equity, or disparities by name. Many studies (61.3%) excluded participants based on demographic, sociocultural, or health factors, primarily due to language proficiency. The new consensus statement includes recommendations for concussion treatment and rehabilitation that rely on an evidence base that has not included SDoH or studies addressing health equity. Researchers are encouraged to design treatment and rehabilitation studies that focus specifically on underrepresented groups to determine if they have specific and unique treatment and rehabilitation needs, whether certain practical modifications to treatment protocols might be necessary, and whether completion rates and treatment adherence and response are similar.

## Introduction

Concussion treatment approaches have evolved considerably over time. Prior approaches included passive “watchful waiting,” vague recommendations to “rest,” or even highly restrictive prescriptions of complete/severe activity and stimulus avoidance until symptoms fully resolved, sometimes referred to as “cocoon therapy”^1^ and sometimes involving a “dark room.”^2^ These approaches were based on precautionary principles, not based on findings from treatment studies and clinical trials,^3^ and some had the potential for negative consequences, including the possibility to *prolong* recovery.^1,4^ Fortunately, the rapid accumulation of concussion treatment studies and clinical trials has led to these earlier approaches being scientifically evaluated and refuted or modified, with the effectiveness of quite different approaches being established.

According to the latest evidence-informed management recommendations from the Concussion in Sport Group (CISG), a multidisciplinary group of international experts who meet every four years to issue updated guidance, strict rest until complete resolution of symptoms is not considered beneficial following sport-related concussion, and instead, early return to physical activity (after 24–48 h) is recommended.^5^ The latest CISG consensus conclusions were informed by numerous systematic reviews, including reviews examining the evidence for prescribed rest or exercise following concussion^6^ and targeted interventions to facilitate concussion recovery.^7^ Taken together, these two systematic reviews examined a substantial body of literature and identified the available clinical science on sport-concussion treatment and rehabilitation.

An important limitation highlighted in one of the systematic reviews was that most concussion treatment studies do not consider ethnicity, socioeconomic status (SES), or social determinants of health (SDoH) that may influence outcomes.^7^ SDoH refer to the environments and conditions in which we are born, raised, educated, live, and work that significantly influence health and well-being.^8–10^ Several SDoH domains and associated subcategories have been identified, including *Economic Stability* (e.g., poverty, ability to afford health care, and housing instability), *Education Access and Quality* (e.g., language and literacy, high school graduation, and enrollment in higher education), *Health Care Access and Quality* (e.g., access to health care including primary care, health literacy, and health and dental insurance), *Neighborhood and Built Environment* (e.g., environmental conditions such as safe air and water, biking and walking accessibility, and crime and violence), and *Social and Community Context* (e.g., experiences of discrimination or racism, parental mental health, and positive versus negative relationships within the family and community).^11,12^ These are overlapping domains, with current and historical distribution of money, power, and resources influencing all SDoH. Social determinants are associated with (1) increased risk for unintentional injuries in preschool age children^13^; (2) prevalence rates of asthma, diabetes, and attention-deficit/hyperactivity disorder (ADHD) in children^14^; (3) early childhood mental health difficulties/concerns^15,16^; and (4) depression and anxiety in adolescents.^17^

Social determinants are causal factors in *health inequity*, which refers to systematic group differences in health status and outcomes that impose significant social and economic costs both to individuals and societies.^18^ *Health equity* means that all people should have fair and equal opportunity to enjoy their full health potential.^19^ Health inequity can be socially patterned across a variety of demographic and sociocultural factors. Disparities in health care access and health outcomes have been reported in association with SES,^20,21^ English language proficiency,^22^ acculturation,^23,24^ and race and ethnicity.^25–27^

There is accumulating evidence showing the clear and expected relevance of SDoH and health equity factors with regard to concussion treatment and rehabilitation. Namely, racial, ethnic, and socioeconomic disparities have been reported with regard to accessing concussion care,^28–30^ recovery time and supports received upon returning to school following the injury,^31,32^ medical follow-up until clearance,^33^ and concussion-related health literacy (i.e., general concussion knowledge, awareness of concussion symptoms, and familiarity with concussion laws).^34–36^ Moreover, a narrative review and content analysis^37^ reported that SDoH-related variables are underrepresented as important, meaningful, or primary variables of interest directly addressed in the clinical literature relied upon to devise the diagnosis, prognosis, and treatment recommendations in the *Centers for Disease Control and Prevention Guideline on the Diagnosis and Management of Mild Traumatic Brain Injury Among Children.*^38^

The recently published systematic reviews conducted by the CISG represent an extraordinary synthesis of a broad and diverse sport-concussion treatment and rehabilitation literature. Leveraging this significant effort from multidisciplinary experts, the purpose of the current study was to conduct a narrative review and content analysis of the studies identified in the two systematic reviews on concussion treatment and rehabilitation,^6,7^ and relied upon to support the CISG practice recommendations.^5^ The goals were to determine the extent to which the available clinical science on concussion treatment and rehabilitation has examined or discussed SDoH or health equity, identify critical knowledge gaps, and provide recommendations to enhance the incorporation of SDoH and health equity in concussion treatment outcome research and clinical care.

## Methods

### Selection of studies and data extraction

We selected sport-related concussion treatment outcome studies that were identified through two systematic reviews.^6,7^ The systematic review on exercise and rest following concussion screened 6,207 records, assessed 81 articles for eligibility, and ultimately included 46 studies.^6^ From those 46 studies, we included 19 on prescribed treatment or rehabilitation, as opposed to studies examining participants who were naturally more or less active following injury (there were 15 treatment studies examining prescribed exercise and there were an additional 4 studies that examined prescribed rest). The systematic review on targeted interventions screened 6,533 records, assessed 154 articles for eligibility, and ultimately included 13 studies.^7^ From those 13 studies, we included 12 for this content analysis (one study was excluded because it did not involve a prescribed form of treatment—it compared recovery time based on self-reported cognitive activity level and 2 studies were duplicates from the exercise and rest review). Taken together, for this content analysis we included and coded a total of 31 studies.

### Data extraction

The research team developed a coding sheet to extract SDoH and the associated subcategories derived from both the Healthy People 2020 (https://wayback.archive-it.org/5774/20220413203948/https://www.healthypeople.gov/2020/topics-objectives/topic/social-determinants-of-health) and Healthy People 2030 (https://health.gov/healthypeople/objectives-and-data/social-determinants-health) websites. These websites list five SDoH domains: *Economic Stability*, *Education Assess and Quality*, *Health Care Access and Quality*, *Neighborhood and Built Environment*, and *Social and Community Context*. The Healthy People 2020 website listed each of these five domains with several key issues and underlying factors within each domain. For instance, a key issue listed under *Social and Community Context* is “social cohesion.” We included each of the key issues and underlying factors as separate variables to code/extract (i.e., whether study authors examined or referenced the SDoH in any way). We then cross-referenced this list with the updated Healthy People 2030 website, which provides a separate web page for each SDoH domain with an “Overview and Objectives” section that has brief text descriptions. Three research team members independently read each text description and extracted any additional key issues or underlying factors that were not included on the Healthy People 2020 website. For example, in the *Economic Stability* domain, the ability to afford health care was listed in Healthy People 2030, but it was not on the Healthy People 2020 list, so it was added to the coding sheet. On the coding sheet, authors determined and indicated whether articles addressed SDoH or not, and, if so, whether the study examined SDoH in an *inferential or intentional* way (e.g., the SDoH variable represented a primary focus or emphasis of the study, or the SDoH variable served as a primary predictor of treatment outcome), or in a *descriptive or demographic* way (e.g., the SDoH variable was summarized as a demographic variable only or mentioned in the discussion section as an area of future study). Coders also indicated whether the study explicitly mentioned (1) SDoH by name or (2) health equity or disparities by name (yes or no).

The coding sheet also included summary information about the study sample, including the sample size, average age, age range, as well as the gender, racial, and ethnic composition of the sample (coders collected the exact sample details or identified that the information was “Not Reported”). The coding sheet also included the study setting and design, whether the study included exclusionary criteria based on demographic, sociocultural, or health factors, whether the study authors indicated any limitations to their study regarding SDoH or health equity, and whether the study authors mentioned future directions/research needs regarding social determinants or health equity. Lastly, coders indicated whether, or the extent to which, each study analyzed or provided information about the following five health equity variables: race, ethnicity, culture/acculturation, SES, and language. Coders could indicate whether the study (1) provided no mention of the variable, (2) included the variable as a demographic category only, or (3) examined the variable in depth (e.g., the health equity variable was a primary variable of interest in the study or outcome results were stratified and analyzed or reported across levels of the variable). The study coding sheet is included in the Supplementary Data S1. Research team members worked in pairs and reviewed the 31 studies. Each study was independently coded by two raters who performed a content analysis by extracting details for each study and completing the coding sheet described above. Discrepancies were resolved by discussion.

One research team member identified when each study began. This was collected to examine whether studies designed and started more recently might be more likely to incorporate SDoH. The year that studies were designed/started was determined using the following two sources: (1) the article itself (i.e., whether authors reported when their study began) and (2) ClinicalTrials.gov (if the study was registered). Information on when the study started was available for all but four studies. For one study, the information available in the article itself and on ClinicalTrials.gov differed (i.e., reported a different year for when the study started in the article compared with what is listed on ClinicalTrials.gov—the sources differed by one year), and for that study we included the earlier year.

### Data analysis and synthesis

The results of this content analysis were summarized descriptively and narratively. Statistical significance testing, quantitative synthesis, or meta-analytic techniques were not used. The percentages of studies that were identified as including the variables of interest (variables from the coding sheet described above) were calculated and summarized descriptively.

## Results

The 31 studies included a total of 2,698 participants (median = 50 participants per study; range 9–456). Most studies were conducted in the United States (*k* = 25; 80.6%), with nearly all remaining studies conducted in Canada (*k* = 5; 16.1%); a single study was conducted at sites in both the United States and Canada (*k* = 1; 3.2%). Studies were started/began between 2007 and 2018, with roughly half (52%) being started since 2015. Studies were published between 2013 and 2022, with slightly more than half (55%) being published since 2019. The average sample age was reported in 27 studies (87.1%) and was 16.2 years (SD = 2.5; median = 15 years). Samples included individuals ranging from 8 to 33 years old. Approximately 2 out of 3 of the included studies (67.9%) were focused on youth (children to older adolescents), with the sample including participants aged 19 or younger. Gender was reported in nearly all studies (*k* = 30; 96.8%). The average percentage of female participants was 47.6% (SD = 19.6; median = 46%; range 0%−100%). Racial composition of the samples was reported in about one in five studies (*k* = 6; 19.4%). The median percentage of study participants identified as white was 81% (range 69%−86.7%; reported in 6 studies). The median percentage of study participants identified as black/African American was 6.6% (range 2.0%−25.0%; reported in 5 studies). The median percentage of study participants identified as Asian was 6.3% (range 3.5%−8.2%; reported in 4 studies). Two studies reported the percentage of participants identified as “other” race (median = 7.9%; range 1.5%−14.3%) and one study reported the percentage of individuals identified as multiracial (8.5%). In absolute terms, the few studies that reported racial composition of their sample included between 1 and 8 participants who identified as black and between 1 and 7 participants who identified as Asian. Ethnic composition of the samples was reported in about one in eight studies (*k* = 4; 12.9%). The median percentage of these four study samples who self-identified as Hispanic was 5.9% (percentages from the four studies were as follows: 3.3%, 3.6%, 8.2%, and 8.5%). Four out of five studies (80.6%) reported neither race nor ethnicity of their sample. In absolute terms, the few studies that reported ethnic composition of their sample included from 1 to 17 participants who identified as Hispanic.

Most studies recruited from specialty concussion clinics (*k* = 24; 74.4%). A few studies recruited from emergency departments (*k* = 3; 9.7%) and collegiate athletics (*k* = 3; 9.7%). One study recruited from a college health center (3.2%). No study recruited exclusively from pediatric primary care or community health centers. One study recruited from numerous settings, which included pediatric primary care; however, only 2 participants (1% of the total sample) were recruited from primary care.^39^ One other study also recruited from several settings, including the community, although it was not reported how many participants were ultimately recruited from the community as opposed to the other settings, which were specialty head injury clinics and emergency departments (and the study did not provide further detail or elaboration on what community setting(s) were used for recruitment).^40^ Study designs included primarily randomized controlled trials (RCT) (*k* = 21; 67.7%). Other designs included prospective cohort (*k* = 5; 16.1%), retrospective case–control (*k* = 2; 6.5%), prospective case–control (*k* = 2; 6.5%), and there was one retrospective cohort study (3.2%). A wide range of treatments and interventions were studied (see Table 1).

**Table 1.** Summary of Studies and Samples Included in the Content Analysis

First author (year published); year started	Country	Setting	Days postinjury	Intervention	N,(Tx n)	Age	%Female	% Hispanic	% Black	% ‘Nonwhite’	Household income
Bailey (2019)^41^; 2014	United States	Specialty clinic	M (SD): 56 (29.33)	Subsymptom threshold exercise	15 (7)	M (SD): 15.8 (1.4)	44%	–	25%	31%	–
Buckley (2016)^42^; 2012	United States	Collegiate sports	1	Cognitive and physical rest	50 (25)	M (SD): Rest 19.8 (1.2); No Rest: 19.4 (1.3)	38%	–	–	–	–
Chan (2018)^43^; 2013	Canada	Specialty clinic	132 (52)	Active rehabilitation	19 (10)	M (SD): 15.5 (1.5)	74%	–	–	–	–
Chizuk (2022)^44^; 2018	United States	Specialty clinic	6.48 (2.2)	Aerobic exercise	51 (31)	M (SD): 15.8 (1.6)	39%	–	–	–	–
Chrisman (2019)^45^; 2016	United States	Specialty clinic	3 weeks to 6 months	Exercise	30 (19)	M (SD): 15.5 (1.6)	57%	3.3%	6.6%	13.2%	$0–60,000 per year: 20%
Clausen (2016)^113^	United States	Specialty clinic	9 weeks	Subsymptom threshold exercise	9 (6)	M (SD): PCS 23 (6); Healthy 21 (3)	100%	–	–	–	–
Congeni (2022)^46^; 2017	United States	Specialty clinic	Treatment: 4.14 Control: 4.5	Head and Neck cooling therapy	55 (28)	M (SD): Treatment 14.7 (1.6); Control: 14.3 (1.4)	49%	3.6%	14.5%	18.1%	–
Gauvin-Lepage (2019)^114^; 2012	Canada	Specialty clinic	30.5 (3.7)	Active rehabilitation	355 (355)	M (SD): 14.3 (2.2)	53%	–	–	–	–
Gibson (2013)^115^; 2007	United States	Specialty clinic	Symptoms <30 days: 19.4 (47.5) Symptoms >30 days: 20.5 (23.3)	Cognitive rest	184 (85)	M (SD): 15.0 (3.3)	28%	–	–	–	–
Haider (2019)^47^; 2013	United States	Specialty clinic	Aerobic: 4.9 (2), Placebo: 4.8 (2) Rest: 4.2 (2)	Subthreshold Aerobic exercise, relative rest, vs. placebo-stretching	130 (52)	M (SD): Aerobic 15.3 (1.6); Placebo 15.4 (1.7); Rest 15.2 (1.4)	47%	–	–	–	–
Howell (2021)^116^; 2018	United States	Specialty clinic	<14 days	Aerobic exercise	37 (17)	M (SD): Exercise 17.2 (2.0); Control: 16.8 (2.2)	41%	–	–	–	–
Kontos (2021)^48^; 2018	United States	Specialty clinic	Treatment: 5.7 (4.0) Control: 6.6 (4.0)	Vestibular rehabilitation	50 (25)	M (SD): Vestibular 15.3 (1.6); Control 15.3 (1.7)	62%	–	–	–	–
Kurowski (2017)^40^; 2013	United States	Specialty clinic	Treatment: 52.30 (19.93) Control: 55.95 (22.16)	Subsymptom threshold exercise	30 (15)	M (SD): Treatment 15.2 (1.4); Control: 15.5 (1.8)	57%	–	–	13.3%	$70,000 and above: 62%
Leddy (2013)^49^	United States	Specialty clinic	Treatment: 65.25 Stretching: 170.75 Control: N/A	Exercise	12 (4)	M (SD): 23.3 (5.3)	67%	–	–	–	–
Leddy (2019a)^50^; 2015	United States and Canada	Specialty clinic	Treatment: 4.9 (2.2) Control: 4.8 (2.4)	Subsymptom threshold exercise	103 (52)	M (SD): Exercise 15.3 (1.6); Stretching 15.4 (1.7)	47%	–	–	–	–
Leddy (2019 b)^51^; 2015	United States	Specialty clinic	Treatment: 4.75 (2.47) Control: 4.50 (2.13)	Subsymptom threshold exercise	54 (24)	M (SD): Treatment 15.1 (1.4); Control: 15.3 (1.4)	0%	–	–	–	–
Leddy (2021)^52^; 2018	United States	Specialty clinic	Treatment: 5.8 (2.3) Stretching: 6.3 (2.4)	Subsymptom threshold exercise	118 (61)	M (SD): Treatment 15.5 (1.4); Stretching 15.9 (1.4)	37%	–	–	–	–
Ledoux (2022)^53^; 2017	Canada	Emergency department	Experimental: 3.3 median hours Control: 3.0 median hours	Progressive physical activity 72 Hours postinjury	456 (227)	M (SD): Experimental 13.3 (2.1); Control 13.3 (2.2)	44%	–	–	–	–
Maerlender (2015)^117^; 2010	United States	Collegiate sports	2 median days	Daily moderate physical exertion	28 (13)	–	72%	–	–	–	–
McCarty (2016)^54^; 2014	United States	Specialty clinic	66.0 (45) median days	Collaborative care	49 (25)	M (SD): 15.0 (1.6)	65%	8.2%	2.0%	24.5%	<$50,000: 20.4%; $50,000–100,000: 26.5%; >$100,000: 46.9%; Unknown: 6.2%
McCarty (2021)^39^; 2017	United States	Specialty clinic	<9 months	Collaborative care	200 (101)	M (SD): 14.7 (1.7)	62%	8.5%	2.5%	17%	<$50,00: 8.5%; $50,000–99,999: 19%; $100,000–150,000: 25.5%; >$150,000: 41.5%; Unknown: 6.0%
Micay (2018)^118^; 2017	Canada	Specialty clinic	<5 days	Structured aerobic exercise	15 (8)	M (SD): Exercise 15.8 (1.2); Control 15.6 (1.0)	0%	–	–	–	–
Reddy (2013)^55^	United States	Specialty clinic	26.18 (36.70)	Amantadine	50 (25)	M (SD): 15.5 (1.4)	56%	–	–	–	–
Reneker (2016)^56^; 2014	United States	Specialty clinic	Treatment: 12.5 (1.6) Control: 11.8 (1.6)	Physical therapy	41 (22)	M (SD): Treatment ∼16.5 (2.9); Control 15.9 (2.9)	39%	–	–	–	–
Schneider (2014)^57^; 2010	Canada	Specialty clinic	Treatment: 53 days (median) Control: 47 days (median)	Cervicovestibular rehabilitation	31 (15)	Median: 15	42%	–	–	–	–
Standiford (2021)^58^; 2016	United States	Emergency department	<48 h	Magnesium	17 (9)	–	–	–	–	–	–
Stumph (2019)^119^; 2015	United States	Specialty clinic	6.0 days (median)	Subsymptom exacerbation early exercise	187 (112)	<10 = 1 (0.5%); 10–12 = 42 (23%); 13–15 = 103 (55%); 16–17 = 37 (20%); >18 = 4 (2%)	45%	–	–	–	–
Thomas (2015)^59^; 2010	United States	Emergency department	<24 h	Strict rest	99 (49)	Median: 13.7	34%	–	–	–	–
Walter (2017)^60^	United States	Collegiate sports	6 months to 3 years	Enzogenol supplementation	42 (–)	–	52%	–	–	–	–
Willer (2019)^61^; 2013	United States	Specialty clinic	Exercise: 4.9 (2.2) Stretching: 4.8 (2.4) Rest: 4.3 (2.0)	Aerobic exercise	151 (52)	M (SD): Exercise 15.3 (1.6); Stretching 15.4 (1.7); Rest 15.4 (1.4)	40%	–	–	–	–
Yao (2020)^120^; 2015	United States	College health center	3.43	Osteopathic manipulative medicine intervention	30 (16)	M: 19.9	39%	–	–	–	–

–, not reported; *N* = total sample size; Tx *n* = sample size of the treatment/intervention group; M = mean; SD = standard deviation; SES, socioeconomic status. The nonwhite classification is used for studies using that term and for all studies represents the total percentage of subjects in the study who were reported as having self-identified race as something other than white.

### Social determinants of health

No study explicitly mentioned “SDoH” by name. The content analysis results related to SDoH domains among the 31 included studies are summarized in Table 2. The relevant quotes from articles that were coded as referencing SDoH are provided in the Supplementary Data S1. Four studies (12.9%) examined an SDoH domain in an inferential/intentional manner.^39,40,45,54^ Of these, three studies examined a single SDoH domain (two of these examined a single subcategory within the domain and the third study examined two subcategories in the domain) and one study examined two SDoH domains in an inferential/intentional manner (examining a single subcategory in each domain). Four additional studies (12.9%) examined a single SDoH domain in a descriptive manner^46,52,53,56^ and one additional study (3.2%) examined two SDoH domains in a descriptive manner.^42^ A greater percentage of studies started more recently tended to include SDoH. Specifically, the following percentages of studies started in various years included SDoH in some capacity: 2007–2010 = 0%; 2012–2013 = 16.7%; 2014–2015 = 28.6%; 2016–2017 = 50%; and 2018 = 25%. Similarly, a greater percentage of more recently published studies included SDoH compared with studies published longer ago. Specifically, the following percentages of studies published in two-year increments included SDoH in some capacity: 2013–2014 = 0%; 2015–2016 = 16.7%; 2017–2018 = 25%; 2019–2020 = 22.2%; and 2021–2022 = 37.5%.

**Table 2.** Summary of Content Analysis Results Regarding Inclusion of Social Determinants of Health

First author (year)	Social determinants of health (SDoH)				
	Economic stability	Education access and Quality	Health care access and Quality	Neighborhood and Built Environment	Social and Community Context
Bailey (2019)^41^	–	Descriptive	Descriptive	–	–
Buckley (2016)^42^	–	–	–	–	–
Chan (2018)^43^	–	–	–	–	–
Chizuk (2022)^44^	–	–	–	–	–
Chrisman (2019)^45^	–	Descriptive	Inferential	–	Inferential
Clausen (2016)^113^	–	–	–	–	–
Congeni (2022)^46^	–	Descriptive	–	–	–
Gauvin-Lepage (2019)^114^	–	–	–	–	–
Gibson (2013)^115^	–	–	–	–	–
Haider (2019)^47^	–	–	–	–	–
Howell (2021)^116^	–	–	–	–	–
Kontos (2021)^48^	–	–	–	–	–
Kurowski (2017)^40^	–	Descriptive	Inferential	–	–
Leddy (2013)^49^	–	–	–	–	–
Leddy (2019a)^50^	–	–	–	–	–
Leddy (2019 b)^51^	–	–	–	–	–
Leddy (2021)^52^	–	–	Descriptive	–	–
Ledoux (2022)^53^	–	–	Descriptive	–	–
Maerlender (2015)^117^	–	–	–	–	–
McCarty (2016)^54^	–	Descriptive	–	–	Inferential
McCarty (2021)^39^	–	Descriptive	Inferential	–	–
Micay (2018)^118^	–	–	–	–	–
Reddy (2013)^55^	–	–	–	–	–
Reneker (2016)^56^	–	Descriptive	–	–	–
Schneider (2014)^57^	–	–	–	–	–
Standiford (2021)^58^	–	–	–	–	–
Stumph (2019)^119^	–	–	–	–	–
Thomas (2015)^59^	–	–	–	–	–
Walter (2017)^60^	–	–	–	–	–
Willer (2019)^61^	–	–	–	–	–
Yao (2020)^120^	–	–	–	–	–

–, not addressed; Inferential, SDoH variable represented in an inferential or intentional way (e.g., a primary focus or emphasis of the study, a primary predictor of outcome); Descriptive, SDoH variable represented in a descriptive or demographic way (e.g., summarized as a demographic variable only, mentioned in the Discussion section as an area of future study, or utilized as a design feature of the study such as might pertain to recruitment, inclusion/exclusion criteria).

No studies examined the *Economic Stability* or *Neighborhood and Built Environment* SDoH domains, either in an inferential or descriptive manner. *Social and Community Context* was examined in an inferential/intentional manner by two studies, with both examining parental mental health. One of these studies examined change in parental fear avoidance over time and also whether parental fear avoidance changed differentially in the treatment and control groups.^45^ The other study, evaluating a collaborative care intervention, assessed parental anxiety and depression, and examined whether these parental mental health outcomes changed over the course of treatment.^54^

The most common SDoH domains represented were *Education Access and Quality*, which was examined exclusively in a descriptive manner in seven studies (see Table 2), and *Health Care Access and Quality,* which was examined in an inferential/intentional manner by three studies and in a descriptive manner in three additional studies. Regarding *Educational Access and Quality,* two studies conducted by the same authors/research team, included the same three subcategories (i.e., *enrollment in higher education* by reporting parental education level, *whether youth have disabilities* by reporting the percentage of their sample with a learning disorder or ADHD, and *whether youth are from low-income families* by reporting annual household income^39,54^). Five studies included a single relevant subcategory. Three of these studies reported, descriptively, whether youth had conditions known to negatively impact academic/educational performance (i.e., ADHD or learning disorder).^41,46,56^ Another study examined *whether youth are from low-income families* by reporting the proportion of the sample with a household income between $0 and $60,000 per year^45^ and another examined *enrollment in higher education* by reporting the proportion of the sample with a primary caregiver with a bachelor’s degree or level of education higher education.^40^

Regarding inferential/intentional coverage of *Health Care Access and Quality,* a subthreshold exercise program was specifically designed to require minimal in-person visits to reduce barriers to accessing health care providers,^45^ another exercise intervention study provided each youth with a portable exercise bike,^40^ which supported access to health care recommendations and prescribed treatment, and a collaborative care study delivered treatment mostly via telehealth with the authors explicitly stating that telehealth reduced transportation barriers and scheduling conflicts.^39^ Descriptive coverage of the *Health Care Access and Quality* domain included a study of treatment outcomes from an existing multidisciplinary concussion clinic with the authors describing their program’s emphasis on providing education and information about concussion,^41^ which serves to increase health literacy, an RCT described the importance of study setting (i.e., community-based clinics vs. hospital-affiliated sites) as relates to differences in access to referrals,^52^ and a treatment program empowered self-management at home,^53^ thus reducing transportation and other barriers to accessing health care.

### Health equity factors

No study examined health equity or disparities directly, such as by explicitly focusing their research on this topic or area of inquiry. Health disparities were described in just one study, and this reference was to prior literature in the study’s discussion section suggesting health care access disparities based on ethnicity and insurance status (private or public).^39^ The content analysis results related to health equity factors among the 31 included studies are summarized in Table 3. No studies examined or reported on culture/acculturation or language. Five studies (16.1%) examined race as a demographic category/sample descriptor. One study (3.2%) examined race in depth by analyzing whether racial identity was associated with treatment response and reported that youth who did and did not return to baseline following treatment did not differ by race.^40^ However, the study reported race as two levels, “white” and “nonwhite,” and only two youth in the treatment condition (and thus included in the described statistical analysis) identified as nonwhite race. Of the six studies that included data on the sample’s race, four (12.9% of the total studies) included ethnicity, but exclusively as a demographic category. No studies examined ethnicity in-depth, such as by investigating whether treatment preferences or outcomes might differ between individuals of various ethnic identities. Moreover, all but one of these studies used race and ethnicity interchangeably (i.e., most studies reported a combined “race/ethnicity” variable or reported the percentage of the sample identifying as Hispanic under the heading “Race”). Three studies (9.7%) examined SES (measured as household income) as a demographic category/sample descriptor^39,45,54^ and one study (3.2%) examined SES in depth by testing whether the treatment and control groups differed based on SES.^40^ Of note, it was the same study that examined race and SES in depth; thus, a single study (3.2% of the included studies on concussion treatment) examined any health equity variables in depth. Moreover, the analyses of race and SES appeared to represent secondary analyses in the study and were not a priori stated goals or primary objectives of the research study.

**Table 3.** Summary of Content Analysis Results Regarding Health Equity Factors, Exclusionary Criteria, and Future Directions

First author (Year)	Health equity factors					Exclusion criteria^b^	Limitations^a^	Future directions^c^
	Race	Ethnicity	SES	Culture	Language			
Bailey (2019)^41^	Demographic	–	–	–	–	–	–	–
Buckley (2016)^42^	–	–	–	–	–	–	–	–
Chan (2018)^43^	–	–	–	–	–	Yes	–	–
Chizuk (2022)^44^	–	–	–	–	–	Yes	Yes	–
Chrisman (2019)^45^	Demographic	Demographic	Demographic	–	–	–	Yes	–
Clausen (2016)^113^	–	–	–	–	–	–	–	–
Congeni (2022)^46^	Demographic	Demographic	–	–	–	Yes	–	Yes
Gauvin-Lepage (2019)^114^	–	–	–	–	–	–	–	–
Gibson (2013)^115^	–	–	–	–	–	–	–	–
Haider (2019)^47^	–	–	–	–	–	Yes	–	–
Howell (2021)^116^	–	–	–	–	–	–	–	–
Kontos (2021)^48^	–	–	–	–	–	Yes	–	–
Kurowski (2017)^40^	In depth	–	In depth	–	–	Yes	Yes	–
Leddy (2013)^49^	–	–	–	–	–	Yes	–	–
Leddy (2019a)^50^	–	–	–	–	–	Yes	–	–
Leddy (2019 b)^51^	–	–	–	–	–	Yes	–	–
Leddy (2021)^52^	–	–	–	–	–	Yes	–	–
Ledoux (2022)^53^	–	–	–	–	–	Yes	–	–
Maerlender (2015)^117^	–	–	–	–	–	–	–	–
McCarty (2016)^54^	Demographic	Demographic	Demographic	–	–	Yes	–	–
McCarty (2021)^39^	Demographic	Demographic	Demographic	–	–	Yes	Yes	–
Micay (2018)^118^	–	–	–	–	–	–	–	–
Reddy (2013)^55^	–	–	–	–	–	Yes	–	–
Reneker (2016)^56^	–	–	–	–	–	–	–	–
Schneider (2014)^57^	–	–	–	–	–	Yes	–	–
Standiford (2021)^58^	–	–	–	–	–	Yes	–	–
Stumph (2019)^119^	–	–	–	–	–	–	–	–
Thomas (2015)^59^	–	–	–	–	–	Yes	–	–
Walter (2017)^60^	–	–	–	–	–	Yes	–	Yes
Willer (2019)^61^	–	–	–	–	–	Yes	–	–
Yao (2020)^120^	–	–	–	–	–	–	–	–

^a^ Studies acknowledged or discussed limitations regarding social determinants or health equity.

^b^ Studies included exclusionary criteria based on demographic, sociocultural, or health factors.

^c^ Studies mentioned future directions/research needs regarding social determinants or health equity.

–, not addressed; SES, socioeconomic status.

### Demographic, sociocultural, or health factors as exclusionary criteria

About 3 out of 5 studies (61.3%) excluded participants based on demographic, sociocultural, or health factors. Many studies excluded participants with limited English proficiency,^44,47,50–52,61^ if there was a “language barrier,”^53^ if children and/or their parent/guardian were non-English speaking,^40,54^ or if consent could not be conducted in English.^59^ These studies did not describe such exclusion as a limitation, such that the generalizability of the reported results might be limited, nor did they report how many potential participants were excluded based on language status.

Multiple studies excluded participants with preinjury health conditions or disabilities, such as a history of developmental disorder,^43,60^ delays,^40,53^ or disability,^59^ or neurodevelopmental conditions, including ADHD^47,51,55,59,61^ or learning disorder.^47,51,59,61^ Some studies excluded any individuals with a psychiatric or psychological disorder,^60^ including anxiety or depression,^47,51,61^ while other studies specified “severe” or “significant” psychological problems^40,46,55^ and others only excluded individuals who were in active treatment and/or prescribed medication for ADHD or psychological disorders.^43,44,50,52^ One study only excluded participants taking two or more medications for ADHD or if medications were recently adjusted.^40^ Furthermore, participants were excluded based on history or active substance use/dependence,^44,48,52,54,55^ acute suicidal ideation,^39,54^ diagnoses of bipolar disorder, psychotic disorders, schizophrenia,^39,54^ posttraumatic stress disorder,^49^ or a “disability that hindered adequate response to concussion symptom severity score.”^58^ Some studies provided explanation for such exclusionary criteria, including citing research that such preinjury health conditions have the potential to influence concussion recovery,^47,50,51,61^ and that certain medications may influence symptom reporting, the central nervous system, autonomic function, “neural adaptation,” or recovery.^44,52,55,57^ One study acknowledged that further research on these preinjury health conditions is needed to address generalizability.^51^ That said, many studies did not provide a rationale for the exclusion nor describe the decision as a study limitation.^40,43,46,48,53,59^

### Study limitations noted regarding social determinants or health equity

The vast majority of articles (k = 27; 87.9%) did not discuss study limitations related to SDoH or health equity. Four studies acknowledged or discussed limitations regarding social determinants or health equity (12.9%).^39,40,44,45^ Two studies acknowledged explicitly that the samples included predominantly individuals who identify as white race and who reported being from a higher SES.^39,40^ One study included a general statement noting that the study was “limited by a lack of diversity.”^45^ Another study acknowledged that the potential influence of SES on treatment adherence was not evaluated.^44^

### Social determinants or health equity as future clinical and research considerations

The vast majority of studies (k = 29; 93.5%) did not explicitly mention SDoH or health equity as frameworks to guide future concussion treatment research. Two studies (6.5%) referenced future clinical or research needs related to social determinants, health equity, or related factors.^46,60^ One study recommended that future research include “more diverse samples to confirm the generalizability” of the study’s findings^60^ and another study, reporting on results from a pilot RCT, stated that the research group was in the process of evaluating the efficacy of the treatment (head and neck cooling therapy) in “a larger, more geographically diverse population.”^46^

## Discussion

This review and content analysis examined studies on sport-related concussion treatment and rehabilitation identified in two major recent systematic reviews^6,7^ that were used to inform the CISG consensus statement on sport-related concussion.^5^ Our results indicate that the consensus statement^5^ includes recommendations for treatment and rehabilitation of sport-related concussion that rely on an evidence base that has not considered SDoH or health equity factors. Put another way, SDoH, health equity, and health disparities have not been studied in relation to concussion treatment and rehabilitation in meaningful ways and have not represented primary frameworks by which past researchers designed and conducted studies or interpreted results. This is a systemic challenge and problem for the research community. Important social determinants, preferred language, sexual orientation, gender identity, psychosocial adversity, and socioeconomic factors (e.g., insurance type, household income, and caregiver education and employment) are also underreported in the broader pediatric clinical trial literature.^62^ Moreover, the concussion treatment literature included in this review is relatively recent (all studies published since 2013 and more than half published since 2019), a time period during which SDoH have been increasingly accepted and acknowledged. However, encouragingly, it appeared that more recently published studies were more likely to have incorporated SDoH in some manner, and hopefully this will continue and accelerate. The clinical and research implications of our findings, including the underrepresentation of SDoH in the concussion treatment and rehabilitation literature, are described below.

### Underrepresentation, race, and ethnicity

The racial composition of the samples was reported in about one in five studies (*k* = 6; 19.4%) and ethnicity was reported less frequently, in about one in eight studies (*k* = 4; 12.9%). Four out of five studies (80.6%) reported neither the racial nor the ethnic composition of their sample. This problem is not unique to concussion treatment literature. Similar results were reported in a recent systematic review of the broader literature related to the diagnosis, treatment, or recovery of sport-related concussion in school-aged athletes, which found that 15.5% of studies reported the racial composition of their sample, 7.6% reported ethnicity, and 84.4% of studies reported neither.^63^ Researchers are encouraged to collect and report racial and ethnic identities of research participants, with an understanding of these labels as social not biological constructs, serving as proxies for privilege or marginalization, and with an understanding of the intersection with other marginalized identities that can compound disparities. Furthermore, the majority of studies appeared to use race and ethnicity interchangeably, which is a limitation and something future studies should be clearer about defining.

Concussion treatment and rehabilitation research to date have not focused on, or sufficiently included, traditionally underrepresented youth, including those who are black, Asian, Hispanic, native, or Aboriginal, or those of low SES. For example, among the few studies in this content analysis that reported ethnicity, the average proportion of the sample identified as Hispanic was 5.9%. All four of these studies were conducted in the United States. According to the 2020 U.S. census data, 18.9% of the U.S. population identifies as Hispanic.^64^ Thus, the average representation in concussion treatment trials was roughly one-third the proportion represented in the population. This finding, again, mirrors a challenge facing the broader health literature. In a study of over 20,000 clinical trials registered on ClinicalTrials.gov, researchers found that minority racial and ethnic groups are underrepresented, with the majority of participants identifying as white and the median combined enrollment of minority race/ethnicity groups (black, Hispanic/Latino, Asian, American Indian, other/multiracial) falling below population proportions based on census expectations.^65^ Relatedly, Indigenous peoples who belong to tribes that are sovereign nations might instead be characterized as belonging to a racial group (i.e., “Native American” or “American Indian”) although this might not be strictly correct.^66^ Researchers should strive toward precision and clarity in collecting and describing participant identities and the use of bias-free language^67^ with due consideration to the preferences of participants. Moreover, in a study examining social determinants and recruitment and retention of pediatric participants with mild traumatic brain injury, white or multiracial participants were more likely to return for a postacute follow-up visit than those who identified as black.^68^ Enrollment and retention challenges include transportation, distance, funds for travel, time away from work for parents, parenting burden and ability to arrange for childcare, English language fluency, lack of engagement with research community, and health literacy, and represent important considerations for concussion treatment and rehabilitation researchers moving forward.

These findings raise moral and ethical concerns regarding fairness and inclusion. On a pragmatic level, significant underrepresentation precludes adequate statistical power for subgroup analyses. The few studies included in this review that reported the racial composition of their sample included between 1 and 8 participants who identified as black and between 1 and 7 participants who identified as Asian. Therefore, these studies were not adequately powered to conduct subgroup analyses to investigate, for example, potential differential treatment response. There will likely be substantially increased costs for trials to be more diverse; however, there are significant societal costs with not addressing disparities^69^ and those costs are unacceptably high.^70^

### Health literacy

An important SDoH for concussion treatment research and clinical practice that has not been well-studied is health literacy. Health literacy involves the ability to access and process basic health information and services to make informed health decisions.^71^ Greater health literacy is associated with greater engagement in health-promoting physical activity.^72^ Populations that are vulnerable to having low health literacy and health care access are those with a lower SES, racial/ethnic minorities, people with limited English proficiency, those with limited education, and people with disabilities,^73–76^ with level of acculturation playing an important role in English language-based health literacy among individuals with limited English proficiency (i.e., higher U.S. acculturation is associated with higher English-language health literacy).^77^ Moreover, limited English language proficiency is likely associated with lower health literacy through structural factors and barriers, such as by health care systems having limited health information (e.g., discharge or follow-up instructions) available in other languages, few if any multilingual health care providers, and/or limited access to medical interpreters. People with low health literacy may be more passive and disempowered in visits with doctors and practitioners and, as a group, are more likely to report that visits with health care providers are not helpful or empowering.^78^ In one study, participants with low health literacy and/or limited English proficiency reported the highest prevalence of poor health and were less likely to attend follow-up appointments.^76^ Disadvantaged social and socioeconomic conditions contribute to lower health literacy, which might be a modifiable risk factor of health disparities.^79^ Other cultural influences regarding the concept of health literacy might be relevant to consider as well, such as potential cultural differences in deference to health care professionals or “authorities” and cultural expectations for patients’ engagement and participation in health care interactions.

### Social determinants and mental health

Social determinants are related to mental health, and mental health is related to clinical outcomes following concussion. Researchers have reported that preinjury mental health problems are associated with increased risk for worse clinical outcomes following sport-related concussions.^80^ Postinjury mental health difficulties can be the focus of treatment, in a collaborative care model, for example, but they can also influence willingness to participate and adherence to exercise-based treatments. That is, individuals with preinjury mental health difficulties or vulnerabilities who sustain a concussion or individuals who experience adverse mental health symptoms following a concussion might benefit from targeted, evidence-supported mental health interventions, such as cognitive behavior therapy.^81,82^

It is important to appreciate that a diverse range of social determinants are associated with psychological distress and mental health difficulties in youth,^17^ including housing insecurity,^83,84^ food insecurity,^83,85–87^ parental mental health problems,^88^ bullying,^89,90^ not feeling safe at school,^91^ neighborhood safety,^92^ and racial discrimination.^93–96^ Some social determinants are encompassed within the framework of adverse childhood experiences, or traumatic events such as neglect and abuse that occur before the age of 18, which have been shown to be associated with head and neck injury and concussion in children.^97^ Thus, the associations between social determinants and mental health, and overlapping factors such as adverse childhood experiences, and their possible influences on concussion treatment outcomes, are important to consider.

### Exercise-based treatment and rehabilitation

Exercise is considered a safe and effective treatment and rehabilitation strategy for concussion.^6^ A broad range of personal, social, and environmental factors are associated with exercise and physical activity in adolescents,^98^ including self-efficacy to be physically active.^99,100^ Moreover, socialization and social learning variables within the family system are important for health-related behaviors—such as whether children and adolescents engage in exercise.^101^ Barriers to participating in physical activity and exercise for adolescents are diverse, including perceived lack of time, influence of peers, concern about neighborhood safety, neighborhood physical disadvantage or lack of green space, and inaccessibility of facilities and the cost of using them.^99,102,103^

An important future direction is to extend exercise-based concussion treatment research to underrepresented groups. It might be necessary to consider health and safety risks in neighborhoods, biking and walking accessibility (sidewalks and bike paths), crime and violence, and environmental conditions as potential mediators of the ability to engage in exercise-based treatment or barriers to following prescribed exercise regimens. For patients and research participants to engage in some exercise protocols prescribed for home-based care, they must have access to specialized equipment, including a treadmill and/or stationary bike, and a heart monitor. This would mean the participant has access to a personal home gym, lives in a community with an amenities center, can access the school gym, or has the means to afford a gym membership. One study included in this review addressed potential barriers to access by providing participants with a portable stationary bike to take home.^40^ Even with the exercise bike at home, participants must have space and it must be in a location conducive to exercise.

Some exercise programs allow participants to engage in physical activity outside without the use of specialized equipment. Future studies could consider potential barriers to exercise, including access to green space and parks, whether neighborhoods have sidewalks to jog, lighted pathways, fields for organized sports, bicycle paths or rack availability, walkability, or if participants felt safe outdoors. Youth in families with a lower SES consider environmental factors such as proximity, cost, and safety to be important for their exercise participation.^104^ Future studies may consider reducing barriers to participation in treatment studies by providing participants with gym memberships, access to the physical therapy department for exercise equipment, loaning portable equipment, implementing a home exercise program that does not require equipment, and offering virtual care. Of course, this also raises related considerations such as availability and adequacy of space, transportation, and access to the internet.

### Specialty concussion clinics

Social determinants might influence, in both positive and negative ways, outcomes from treatment and rehabilitation for concussion. The personal background and current life experience of parents and caregivers of injured student athletes are important to consider in research and clinical practice conducted in specialty clinics. Examples include parental education, occupation, health literacy, income, and mental health. Patients presenting to specialty clinics are not representative of the population of youth who sustain concussions, and they tend to have longer or more complicated recoveries than those who do not seek specialty care. There are also disparities in the availability and access to specialty concussion care that limit the generalizability of the findings from these clinics. Youth with public insurance or no insurance have less access to specialty care compared with youth with private insurance.^105^ Concussion clinics, compared with emergency departments, see more white patients.^106^ Patients at concussion clinics are more likely to have private insurance than patients who receive care in the emergency department and they are less likely to have Medicaid or to be self-pay.^106^ Parents who are more educated, have greater health literacy, and who have greater financial resources, combined with excellent health insurance and convenient access to specialty care at academic medical centers, are advantaged regarding the initial access, follow-through, and completion of treatment and rehabilitation for their children. In contrast, children with parents who have lower health literacy, less education, lower SES, lower quality insurance, and/or limited English language fluency are disadvantaged regarding accessing and completing treatment.

### Single-session interventions

Researchers and clinicians should try to move, work around, or dismantle barriers to research participation and obstacles to clinical care. There are opportunities for innovation in the treatment and rehabilitation of concussions that might address, at least in part, certain barriers to access and health care inequities. Clinical researchers might be able to develop on-demand, web-based, single-session interventions. Single-session interventions have been used to promote better emotional health among adolescents.^107–109^ In concussion care, they could be developed for (1) recommendations for rest and activity pacing during the few first few days following injury, (2) practical strategies for promoting effective and durable return to school, (3) improving sleep, (4) steps for gradual resumption of exercise, and (5) managing stress, worry, and anxiety.

### Limitations

This content analysis examined treatment and rehabilitation studies that were identified in two recent systematic reviews that had inclusion criterion stipulating that more than 50% of the study sample had sustained a concussion via a sports-related mechanism. There are important potential differences between sport-related and other mechanisms of injury (such as fights, assaults, falls, or motor vehicle collisions). It is possible that SDoH might be incorporated or examined differently in studies including predominantly other mechanisms of injury. This review is limited to the literature relating to sport-related concussion and our findings should not be considered generalizable to the broader literature on pediatric mild traumatic brain injury. Furthermore, given the disparities accessing specialty health care, focusing on studies that include samples of predominantly (>50%) sport-related concussions and that predominantly recruit from specialty concussion programs likely contributes to the findings herein that so few of the studies included racially or ethnically diverse samples. In addition, another inclusion criterion from the source reviews that were used to identify the studies for this content analysis was that studies were published in English. Thus, studies published in other languages would include more diverse samples in terms of culture and primary language.

## Conclusions

In recent years, considerable progress has been made to improve concussion treatment and rehabilitation and to establish its safety and efficacy. The research to date has been done mostly in specialty clinics, with predominately white and presumably well-resourced families. The published studies that we reviewed do not support the feasibility or efficacy of concussion treatment and rehabilitation strategies for marginalized, underrepresented, and/or underresourced youth, such as those with limited English language proficiency, those experiencing poverty or basic needs insecurity, or those living in rural areas with limited access to health care.

There is a need for clinical researchers to design treatment and rehabilitation research such that large studies attempt to enroll and complete participants who have been, to date, underrepresented so that we can determine if they have specific and unique treatment and rehabilitation needs, whether certain practical modifications to treatment protocols might be necessary, and whether their completion rates and treatment responses are similar. At present, clinicians and researchers cannot assume that these specific treatment and rehabilitation strategies are feasible and efficacious for all youth, and there is a pressing need for targeted studies with underrepresented and marginalized groups. These studies will be time-consuming, resource-heavy, and difficult to complete. They will require careful planning, and some of the work will need to be pilot studies and feasibility studies. Research methodologies that have not been widely applied in concussion research, such as community-based participatory research, which includes the community’s input on what research questions to ask and what is considered important to the community, as opposed to a “top down” approach as is the norm, will likely prove beneficial. Future studies will also benefit from clinicians and researchers who themselves come from marginalized or underrepresented communities and are proficient in languages in addition to English, alongside scientist-researcher pipeline programs that support investigators with lived experience, who may have better access to, and trust of, target populations to conduct this much-needed work.^110–112^

Some people are at a disadvantage with respect to their health and health care due to social, economic, geographic, political, structural, or systemic reasons. We aspire for all individuals in need to have access to potentially beneficial treatments that are being developed and studied. We want to ensure fair and diverse representation among treatment study participants. Without adequate representation in large studies, and a series of smaller studies targeting underrepresented groups, it is not possible to confidently conclude which treatments work for whom.

## Transparency, Rigor, and Reproducibility Summary

**FIG. 1.**
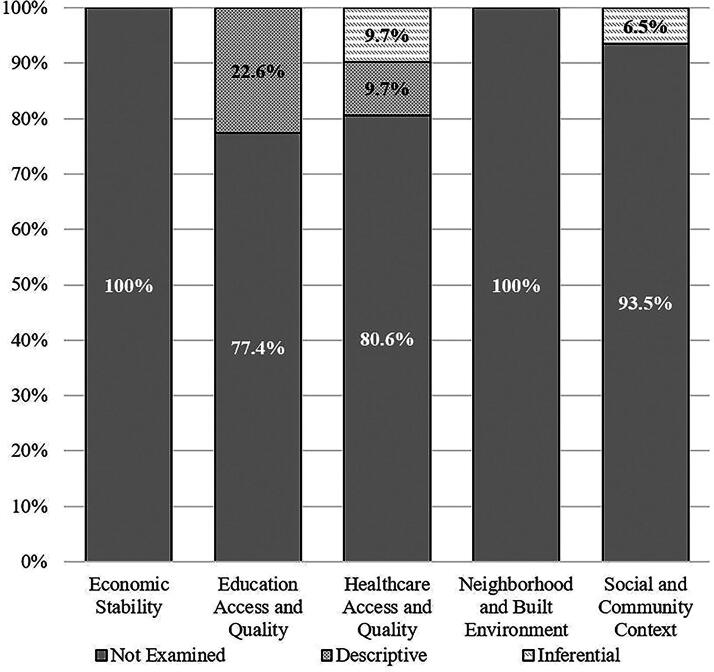
Proportion of studies examining social determinants of health.

**FIG. 2.**
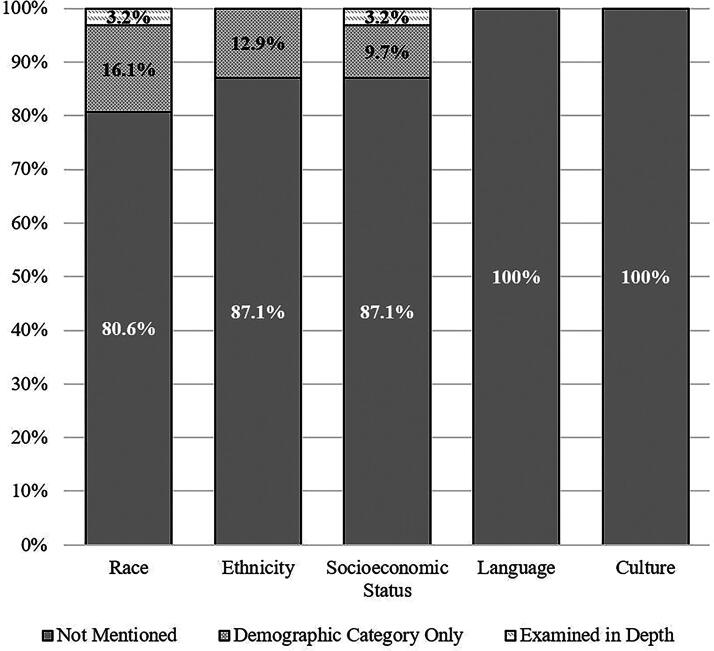
Proportion of studies examining health equity variables.

This review was not preregistered. We conducted a secondary narrative review and content analysis of 31 studies included in two systematic reviews^6,7^ that were part of the CISG effort to develop a consensus statement.^5^ The coding sheet for our content analysis was developed by the authors, adapted from prior work,^37^ and it is provided in the online Supplementary Data S1. Two coauthors independently coded every article and discrepancies were resolved by discussion. The results of this content analysis (i.e., the study coding results) are presented in Tables 2 and 3, Figures 1 and 2, and in the online Supplementary Data S1. Specific quotes from each article that were coded are provided in the Supplementary Data S1. This article will be published under a Creative Commons Open Access license, and upon publication will be freely available at https://www.liebertpub.com/loi/neu.
