# Supplementary material for: Social Determinants of Health and Health Equity in the Treatment and Rehabilitation of Sport-Related Concussion: A Content Analysis of Intervention Research and Call-To-Action
Source: J Neurotrauma. 2024 Oct 21;41(19-20):2201–18. doi: 10.1089/neu.2023.0550 (PMC11564856; doi:10.1089/neu.2023.0550)
Supplement: Supplementary Data S1 [file neu.2023.0550_supplementarymaterialsrevised.docx]

**Online Supplementary Material**

**Social Determinants of Health and Health Equity in the Treatment and Rehabilitation of Sport-Related Concussion: A Content Analysis of Intervention Research and Call-To-Action**

Nathan E. Cook, Ph.D., Department of Physical Medicine and Rehabilitation, Harvard Medical School, Boston, MA; Mass General for Children Sports Concussion Program, Waltham, MA; & Department of Physical Medicine and Rehabilitation, Spaulding Rehabilitation Hospital, Charlestown, MA USA

Alicia Kissinger-Knox, Psy.D., Department of Physical Medicine and Rehabilitation, Harvard Medical School, Boston, MA; Department of Physical Medicine and Rehabilitation, Spaulding Rehabilitation Hospital, Charlestown, MA USA; Concussion Research Program; Spaulding Hospital Cambridge, Cambridge, MA, USA

Ila A. Iverson, Department of Global Public Health, Karolinska Institutet, Stockholm, Sweden

Katie Stephenson, Ph.D., College of Osteopathic Medicine, University of New England, Biddeford, ME

Marc A. Norman, Ph.D., Department of Psychiatry, UC San Diego School of Medicine, San Diego, CA

Amy A. Hunter, MPH, Ph.D., Department of Public Health Sciences, University of Connecticut School of Medicine, Farmington, CT; Department of Pediatrics, University of Connecticut School of Medicine, Farmington, CT; Injury Prevention Center, Connecticut Children’s Medical Center and Hartford Hospital, Hartford, CT

Altaf Saadi, MD, MSc, Department of Neurology, Harvard Medical School, Boston, MA; Department of Neurology, Massachusetts General Hospital, Boston, MA

Grant L. Iverson, Ph.D., Department of Physical Medicine and Rehabilitation, Harvard Medical School, Boston, MA; Department of Physical Medicine and Rehabilitation, Spaulding Rehabilitation Hospital and the Schoen Adams Research Institute at Spaulding Rehabilitation, Charlestown, MA; & Mass General for Children Sports Concussion Program, Waltham, MA; USA

| **Contents** | **Page** |
| --- | --- |
| Supplementary Table 1. Study Coding Sheet | 2 |
| Supplementary Table 2. Quotes from the Articles Relating to Social Determinants of Health | 4 |

**Supplementary Table 1. Study Coding Sheet**

| **First Author:** | **PMID:** | **Coder:** |
| --- | --- | --- |

| **Study/Sample Information** | |
| --- | --- |
| Sample size (N): |  |
| Mean age, SD: |  |
| Age range: |  |
| Gender composition: |  |
| Racial composition: |  |
| Ethnic composition: |  |
| Setting: |  |
| Study Design: |  |

| **Social Determinants Of Health Domains And Subcategories** | | **Did study address?** | | | | |
| --- | --- | --- | --- | --- | --- | --- |
| **Economic Stability: Any of the subcategories?** | | **Inferential** | | **Descriptive** | | **No** |
| Employment security / stable employment | | Inferential | | Descriptive | | No |
| Food insecurity | | Inferential | | Descriptive | | No |
| Ability to afford healthy food | | Inferential | | Descriptive | | No |
| Ability to afford health care | | Inferential | | Descriptive | | No |
| Ability to afford childcare | | Inferential | | Descriptive | | No |
| Housing instability | | Inferential | | Descriptive | | No |
| People with disabilities/injuries and their ability to work | | Inferential | | Descriptive | | No |
| Poverty | | Inferential | | Descriptive | | No |
| Employment programs / career counseling | | Inferential | | Descriptive | | No |
| **Education Access and Quality: Any of the subcategories?** | | **Inferential** | | **Descriptive** | | **No** |
| Early childhood education and development | | Inferential | | Descriptive | | No |
| Enrollment in higher education | | Inferential | | Descriptive | | No |
| High school graduation | | Inferential | | Descriptive | | No |
| Language and literacy | | Inferential | | Descriptive | | No |
| Whether children are from low-income families | | Inferential | | Descriptive | | No |
| Whether children have disabilities | | Inferential | | Descriptive | | No |
| Whether children experience social discrimination (i.e. bullying) | | Inferential | | Descriptive | | No |
| Whether children live in places with poorly performing schools | | Inferential | | Descriptive | | No |
| Whether families can afford to send their children to college | | Inferential | | Descriptive | | No |
| **Health Care Access and Quality: Any of the subcategories?** | | **Inferential** | | **Descriptive** | | **No** |
| Access to health care | | Inferential | | Descriptive | | No |
| Access to primary care | | Inferential | | Descriptive | | No |
| Health literacy | | Inferential | | Descriptive | | No |
| Health and/or dental insurance | | Inferential | | Descriptive | | No |
| Ability to afford health care services and medication | | Inferential | | Descriptive | | No |
| Access to health care service recommendations/referrals | | Inferential | | Descriptive | | No |
| Access to preventative care | | Inferential | | Descriptive | | No |
| Access / transportation to healthcare providers | | Inferential | | Descriptive | | No |
| **Neighborhood and Built Environment: Any of the subcategories?** | | **Inferential** | | **Descriptive** | | **No** |
| Access to foods that support healthy eating patterns | | Inferential | | Descriptive | | No |
| Crime and violence | | Inferential | | Descriptive | | No |
| Environmental conditions (unsafe air or water) | | Inferential | | Descriptive | | No |
| Quality of housing | | Inferential | | Descriptive | | No |
| Racial residential segregation | | Inferential | | Descriptive | | No |
| Other health and safety risks in neighborhood | | Inferential | | Descriptive | | No |
| Workplace health and safety risks | | Inferential | | Descriptive | | No |
| Biking and walking accessibility (sidewalks and bike paths) | | Inferential | | Descriptive | | No |
| **Social and Community Context: Any of the following subcategories?** | **Inferential** | | **Descriptive** | | **No** | |
| Civic participation | Inferential | | Descriptive | | No | |
| Discrimination / Racism | Inferential | | Descriptive | | No | |
| Parental incarceration | Inferential | | Descriptive | | No | |
| Social cohesion | Inferential | | Descriptive | | No | |
| Unsafe neighborhoods | Inferential | | Descriptive | | No | |
| Trouble affording things they need | Inferential | | Descriptive | | No | |
| Societal attitudes and norms (i.e., racism, distrust of government) | Inferential | | Descriptive | | No | |
| Depression or anxiety in family caregivers | Inferential | | Descriptive | | No | |
| Positive versus negative relationships at home | Inferential | | Descriptive | | No | |
| Positive versus negative relationships at work | Inferential | | Descriptive | | No | |
| Positive versus negative relationships in community | Inferential | | Descriptive | | No | |

CODER NOTE: Inferential = the SDoH variable was included/investigated in an inferential or intentional way (e.g., the SDoH variable represented a primary focus or emphasis of the study, or a primary predictor of outcome); Descriptive = the SDoH variable was included/investigated in a descriptive or demographic manner (e.g., the SDoH was summarized as a demographic variable only, mentioned in the discussion as an area of future study, or utilized as a design feature in the study, such as recruitment, inclusion/exclusion criteria)

|  |  |  |
| --- | --- | --- |
| Did the study explicitly mention “social determinants of health” by name? | Yes | No |
| Did the study explicitly mention health equity or disparities by name? | Yes | No |

| **Health Equity Variables** |  | | |
| --- | --- | --- | --- |
| Race | No mention | Demographic category only | Examined in depth |
| Ethnicity | No mention | Demographic category only | Examined in depth |
| Culture | No mention | Demographic category only | Examined in depth |
| Socioeconomic Status | No mention | Demographic category only | Examined in depth |
| Language | No mention | Demographic category only | Examined in depth |

| **Exclusionary Criteria** | | |
| --- | --- | --- |
| Were participants excluded based on demographic or sociocultural or health factors? | Yes | No |
| **Limitations** | | |
| Any limitations regarding social determinants or health equity discussed? | Yes | No |
| **Future Directions** | | |
| Any future directions/research needs regarding social determinants or health equity discussed? | Yes | No |

**Comments/Notes**

*Please include any comments or notes from the article. Please elaborate on any “yes” responses or questions, include the page numbers from the study, and consider copying the actual text excerpt.*

**Supplementary Table 2. Quotes from the Articles Relating to Social Determinants of Health (SDOH) organized alphabetically by first author for each SDoH Domain**

| **First**  **Author** | **Year** | **PMID** | **SDoH Domain** | **SDoH Subcategory** | **Page**  **Number** | **Article**  **Section** |  |
| --- | --- | --- | --- | --- | --- | --- | --- |
| Bailey | 2019 | 31479082 | Health Care Access and Quality | Health Literacy | 382 | Discussion |  |
|  | Quote: “In that sample*,* ***all individuals were provided education regarding concussion and expected outcome*** before engaging in some form of active rehabilitation.” | | | | | | |
| Bailey | 2019 | 31479082 | Education Access and Quality | Whether Children Have Disabilities | 380 | Results |  |
|  | Quote: “Only a minority of participants in the total sample reported ***a history of ADHD/LD*** (12%)” | | | | | | |
| Buckley | 2016 | 26394292 | -- | -- | -- | -- |  |
| Chan | 2017 | 28989074 | -- | -- | -- | -- |  |
| Chizuk | 2022 | 35482774 | -- | -- | -- | -- |  |
| Chrisman | 2019 | 31316446 | Education Access and Quality | Whether Children are from Low-Income Families | 6 | Results |  |
|  | Table 1 reports whether participants have a ***household income of $0-60,000*** per year. | | | | | | |
| Chrisman | 2019 | 31316446 | Health Care Access and Quality | Access / Transportation to Healthcare Providers | 2 | Introduction |  |
|  | Quote: “Currently no exercise treatments have been designed for concussion that could be completed with minimal in-person visits and therefore be more easily disseminated. We proposed to address this gap, adapting a pre-existing exercise intervention for concussion to be ***delivered with only two in-person visits, and utilizing weekly check-ins via phone contact with the youth***, as has been used effectively in previous studies.” | | | | | | |
| Chrisman | 2019 | 31316446 | Social and Community Context | Depression or Anxiety in Family Caregivers | 2 / 7 | Introduction/  Results |  |
|  | Quote: “***Pediatric specific models of fear-avoidance include the important role of parent behavioral and psychological responses to the child’s pain experience***, recognizing that the child’s own experiences develop within the familial context.”  Quote: “***Parental fear-avoidance*** (FOPQ-P) significantly declined overall (p = 0.0096), but was not different by treatment group (b = −0.0031, p = 0.99).” | | | | | | |
| Clausen | 2016 | 26098254 | -- | -- | -- | -- |  |
| Congeni | 2021 | 34009790 | Education Access and Quality | Whether Children Have Disabilities | 345 | Table 2 |  |
|  | Table 2 reports the percentage of ***participants with learning disabilities and ADHD*** | | | | | | |
| Gauvin-Lepage | 2018 | 30045216 | -- | -- | -- | -- |  |
| Gibson | 2013 | 23758286 | -- | -- | -- | -- |  |
| Haider | 2019 | 31105634 | -- | -- | -- | -- |  |
| Howell | 2021 | 33856860 | -- | -- | -- | -- |  |
| Kontos | 2021 | 34450120 | -- | -- | -- | -- |  |
| Kurowski | 2017 | 27120294 | Education Access and Quality | Enrollment in Higher Education | 84 | Results |  |
|  | Table 1: Reports primary caregiver education (***with Bachelor degree or higher***). | | | | | | |
| Kurowski | 2017 | 27120294 | Health Care Access and Quality | Access to Health Care Service Recommendations/ Referrals | 82 / 86 | Methods/ Discussion |  |
|  | Quote: “Individuals in the cycling group were given the ***same portable exercise bike used for study assessments to use at home***. The bike was returned after study completion. Participants were asked to complete the cycling program 5 to 6 days per week at home at 80% of the duration that exacerbated symptoms during the interval and assessment visits.”  Quote: “After the initial screening of participants, 136 participants met full eligibility criteria and approximately 22% enrolled in the study. Most commonly, families were not interested in participation or ***reported that the time commitment was too much***. Individuals who refused participation did not always provide a clear reason, but 13% of this group reported sufficient recovery as to not justify the need for participation in a study.” | | | | | | |
| Leddy | 2013 | 23249769 | -- | -- | -- | -- |  |
| Leddy | 2019a | 30715132 | -- | -- | -- | -- |  |
| Leddy | 2019b | 30239422 | -- | -- | -- | -- |  |
| Leddy | 2021 | 34600629 | Health Care Access and Quality | Access to Health Care Service Recommendations/ Referrals | 797 | Discussion |  |
|  | Quote: “This study also aimed to evaluate generalisability of subthreshold aerobic exercise by ***expanding the initial RCT conducted in community-based sports medicine clinics to hospital-affiliated sites where referrals typically come from primary care physicians or the emergency department***.” | | | | | | |
| Ledoux | 2022 | 34836880 | Health Care Access and Quality | Access to Health Care | 276 | Discussion |  |
|  | Quote: “The intervention ***did not require participants to visit a subspecialty clinic, but rather, empowered self-management at home, which is more generalizable and feasible***. However, the effectiveness of this early intervention does not preclude the possibility of even greater gains if children/youth, especially those at risk of PPCS, are also seen in a subspecialty clinic.” | | | | | | |
| Maerlender | 2015 | 26230745 | -- | -- | -- | -- |  |
| McCarty | 2016 | 27624513 | Education Access and Quality | Enrollment in Higher Education | 5 | Results |  |
|  | Table 1: Provides parental education, including high school graduate or less, ***some college, college graduate, or graduate school***. | | | | | | |
| McCarty | 2016 | 27624513 | Education Access and Quality | High School Graduation | 5 | Results |  |
|  | Table 1: Provides parental education, including ***high school graduate or less***, some college, college graduate, or graduate school. | | | | | | |
| McCarty | 2016 | 27624513 | Education Access and Quality | Whether Children Have Disabilities | 5 | Results |  |
|  | Table 1: Provides preinjury health concerns, including ***previous ADHD or LD diagnosis, history of anxiety or depression***, history of previous concussion, and history of headache. | | | | | | |
| McCarty | 2016 | 27624513 | Social and Community Context | Depression or Anxiety in Family Caregivers | 6 | Discussion |  |
|  | Quote: “No significant group differences were observed over time for anxiety or ***parental mental health***.” | | | | | | |
| McCarty | 2021 | 33635325 | Education Access and Quality | Enrollment in Higher Education | 7 | Results |  |
|  | Table 1: Provides parental education, including high school graduate or less, ***some college, Associate degree, Bachelor’s degree, Graduate school, or other***. | | | | | | |
| McCarty | 2021 | 33635325 | Education Access and Quality | High School Graduation | 7 | Results |  |
|  | Table 1: Provides parental education, including ***high school graduate or less***, some college, Associate degree, Bachelor’s degree, Graduate school, or other. | | | | | | |
| McCarty | 2021 | 33635325 | Education Access and Quality | Whether Children Have Disabilities | 3 | Methods |  |
|  | Quote: “Adolescents were excluded from the study if they had spinal cord or other severe injuries, had a diagnosis of schizophrenia or psychosis, or presented with active, acute suicidal ideation. Patients with prior concussion and/or ***other preexisting psychological disorders were considered eligible***.”  Table 1 reports percentage of youth in the sample with ***prior diagnoses of learning disability*** | | | | | | |
| McCarty | 2021 | 33635325 | Health Care Access and Quality | Access to Healthcare | 10 | Discussion |  |
|  | Quote: “The telehealth delivery was a unique aspect that offered adolescents and families the ***opportunity to receive care at their own convenience without the typical barriers of transportation and more limited scheduling***.” | | | | | | |
| McCarty | 2021 | 33635325 | Health Care Access and Quality | Access / Transportation to Healthcare Providers | 10 | Discussion |  |
|  | Quote: “The telehealth delivery was a unique aspect that offered adolescents and families the ***opportunity to receive care at their own convenience without the typical barriers of transportation and more limited scheduling***.” | | | | | | |
| Micay | 2018 | 30018795 | -- | -- | -- | -- |  |
| Reddy | 2013 | 22613947 | -- | -- | -- | -- |  |
| Reneker | 2017 | 28211600 | Education Access and Quality | Whether Children Have Disabilities | 2010 | Methods |  |
|  | Quote: “Patients with a history of previous concussion(s), ***learning disorders, and attention deficit hyperactivity disorder (ADHD) were included***.” | | | | | | |
| Schneider | 2014 | 24855132 | -- | -- | -- | -- |  |
| Standiford | 2021 | 33243533 | -- | -- | -- | -- |  |
| Stumph | 2019 | 31599706 | -- | -- | -- | -- |  |
| Thomas | 2015 | 25560444 | -- | -- | -- | -- |  |
| Walter | 2017 | 28452602 | -- | -- | -- | -- |  |
| Willer | 2019 | 31377190 | -- | -- | -- | -- |  |
| Yao | 2020 | 32766808 | -- | -- | -- | -- |  |

Note. -- = Not addressed
